# Editorial: Implications of Ferroptosis-Related Genes to the Genetics of Cancer Development

**DOI:** 10.3389/fgene.2022.952340

**Published:** 2022-07-22

**Authors:** Manshan Li, Lei Huang, Ying Li, Lianxiang Luo

**Affiliations:** ^1^ The First Clinical College, Guangdong Medical University, Zhanjiang, China; ^2^ Department of Molecular Cell and Cancer Biology University of Massachusetts Medical School, Worcester, MA, United States; ^3^ Department of Molecular Medicine, UT Health San Antonio, San Antonio, TX, United States; ^4^ The Marine Biomedical Research Institute, Guangdong Medical University, Zhanjiang, China; ^5^ The Marine Biomedical Research Institute of Guangdong Zhanjiang, Zhanjiang, China

**Keywords:** ferroptosis, cancer, lncRNAs, ferroptosis-related genes, traditional Chinese medicine

Ferroptosis is a unique type of cell death, distinct from apoptosis, which refers to an iron-dependent cell death caused by the overload of lipid peroxides on cell membranes (Dixon et al., 2012). Current cancer treatment strategies selectively eliminate cancer cells without harming normal cells. Targeting ferroptosis may provide an alternative strategy for cancer therapy to inhibit tumor growth (Chen et al., 2021; Lei et al., 2022). Studies have shown that multiple cancer-related signaling pathways control ferroptosis in cancer cells, and ferroptosis is also involved in the activity of multiple tumor suppressors (Lei et al., 2022). In addition, some cancer cells rely on ferroptosis defense systems and oxidative stress conditions to survive, and disrupting these ferroptosis defenses can be fatal to cancer cells while leaving normal cells unharmed (Mao et al., 2021). At the same time, the unique metabolism of cancer cells, the high load of reactive oxygen species (ROS), and their specific mutations make cells inherently susceptible to ferroptosis, thus exposing the therapeutic vulnerability of some cancers (Wu et al., 2019). Furthermore, it has been found that oncogene-induced ferroptosis resistance can be mediated by downstream effectors, which can be targeted to reverse ferroptosis resistance and play a strong tumor inhibition effect (Yi et al., 2020). Recent research also suggests that EMT is thought to call into being cancer stem cells, leading to transfer diffusion (Yang et al., 2020), and EMT signaling can promote ferroptosis. Increased CD44-dependent iron endocytosis promotes iron-dependent demethylase activity, which promotes the expression of EMT signaling-related genes, thus making breast cancer cells sensitive to ferroptosis (Müller et al., 2020). Ferroptosis has a wide range of effects on cell metabolism and has many targets in cancer. In addition, various clinical drugs and herbal extracts can induce ferroptosis in cancer cells (Xu et al., 2021). Similarly, ferroptosis-related genes and lncRNA may become potential biomarkers that provide new anti-cancer treatment strategies. Our Research Topic included one review and fifteen original research articles related to Ferroptosis-related genes, lncRNA, and drugs, which may be potential targets for reversing cancer drug resistance and targeted therapy.

LncRNAs are considered to be major regulators of various cellular processes, and their dysregulation is implicated in all features of cancer (Balihodzic et al., 2021). More and more evidence confirmed the importance of lncRNA in regulating ferroptosis (Balihodzic et al., 2022). LncRNAs can regulate the expression of GPX4 and induce lipid peroxidation of the cell membrane through sponge miRNAs, resulting in the ferroptosis of cancer cells (Zuo et al., 2022). We included four ferroptosis-related long non-coding RNA articles. Jiang et al. explored the critical role of ferroptosis-related long non-coding RNAs in the prognosis of head and neck squamous cell carcinoma (HNSCC). They identified ferroptosis-related lncRNAs as independent risk factors for predicting the overall survival outcome of HNSCC patients. In addition, the correlation between patient risk score and PD-1/PD-L1 also revealed the role of the prognosis-related FElncRNA signature (PFLS) in immunotherapy. Zhou et al. also provided ferroptosis-related long non-coding RNA (FRlncRNA) signature to improve the prognostic prediction of ccRCC, suggesting that FRlncRNAs may be a molecular biomarker and therapeutic target of ccRCC. The combination of immunotherapy and targeted therapy can effectively prolong the survival rate of hepatocellular carcinoma (HCC). The FRlncRNAs identified by Zhang et al. have reasonable specificity and sensitivity, which is conducive to immunotherapy and targeted therapy for HCC patients. Li et al. constructed a prognostic model based on 8 FRlncRNAs to provide prognostic assessment for OSCC patients and analyzed differences in immune cells, function, immune checkpoint, and m6A expression between high-risk and low-risk groups. These FRlncRNAs may become novel biomarkers for the treatment of OSCC.

Inducing ferroptosis is a promising strategy for cancer treatment. Finding novel biomarkers of ferroptosis with the good predictive ability and therapeutic targets will effectively reverse drug resistance in cancer. Wang et al. found that a ferroptosis-related gene signature predicts the prognosis of lung adenocarcinoma. Patients in the low-risk group with a better prognosis benefit from enhanced anti-tumor immunity, and this prognostic feature may be a reliable tool for risk stratification in lung adenocarcinoma patients. Similarly, Guan et al. also developed and tested a risk assessment model of genes associated with ferroptosis in sarcomas, in which SLC7A11, FANCD2, CISD1, and ATP3MC3 will be used as new markers to identify patients who are likely to receive adequate ferroptosis induction therapy or combined immunotherapy. A prognostic model based on nine prognostic FRGS was established. Yan et al. established a ferroptosis-related prognostic model to screen patients with low-grade glioma (LGG) for sensitivity to chemotherapy and immunotherapy. The prognostic model is closely related to autophagy and hypoxia. Moreover, this model is helpful for diagnosis, treatment, and prognosis prediction. Cai et al. established ferroptosis-related genes prognostic index (FRGPI) based on HMOX1, TFRC, JUN, and SOCS1, which may help distinguish immune and molecular characteristics and accurately distinguish immune and molecular characteristics predict clinical outcomes, temozolomide resistance, and ICI response in gliomas. In addition, fifteen potential small-molecule compounds were predicted based on FRGPI, which provides a reference for finding effective drugs for treating glioma. Such a systematic assessment of FRGPI in glioma patients may contribute to anti-cancer therapies based on ferroptosis. The study of Zhu et al. also found 14 FRGS, including HMOX1, PEBP1, KEAP1, and LPCAT3. They reveal ferroptosis’s vital role in metastatic tumors by exploring the relationship between ferroptosis and BCBM expression, prognosis, immune response, and drug sensitivity. Interestingly, both pyroptosis and ferroptosis affect the progression and treatment of pancreatic cancer. Yu et al. established a pyroptosis-ferroptosis scoring system based on cell death profiles for pancreatic adenocarcinoma (PAAD) patients. Moreover, the P-F score can predict the outcome and response of chemotherapy drugs or immunotherapy in patients with PAAD. Han et al. found that novel characterization of ferroptosis-related genes driven by CNV can be used to predict the survival of AML. Additionally, CNV-driven DE-FRG may be related to cell cycle disorders and inflammatory immune responses during the disease process. This prognostic model provides potential targets and is helpful for clinical analysis and diagnosis of the AML. ACSL4 is associated with amino acids, lipid synthesis, and lipid peroxide-dependent ferroptosis. Yu et al. explored the expression and prognosis of ACSL4 in pan-cancer and found that ACSL4 plays an essential role in the recruitment and regulation of immune-infiltrating cells in cancer. In addition, the mechanism of ACSL4 in cancer may be closely related to the IGF signaling pathway. IGF-1 receptor (IGF-1R) inhibitors can prevent the binding of IGF-1 to IGF1R, inhibit the PI3K/Akt pathway, and exert anti-tumor activity. ACSL4 may be a potential prognostic immunotherapy biomarker and a potential target for cancer therapy. The study of Ke et al. comprehensively analyzed the potential mechanism and prognostic role of ferroptosis-related genes in hepatocellular carcinoma and found that FTL is a crucial regulator of ferroptosis in HCC. The high expression level of FTL is a poor predictor of survival, and FTL plays a role as an independent prognostic and diagnostic factor for HCC. Finally, Guang et al. analyzed the multiple omics differences among cancer cell lines with high and low ferroptosis scores and constructed a ferroptosis-related model, providing insights into the treatment of various malignancies and targets for cancer drugs.

Ferroptosis can be triggered by small external molecules or drugs (Liang et al., 2019). We have included an article about small molecules in Traditional Chinese medicine to introduce the relevant content. Feng et al. found that Nobiletin can induce ferroptosis in melanoma cells and play an anti-tumor role. Their results showed that Nobiletin induces ferroptosis by modulating the GSK3β -mediated Keap1/Nrf2/HO-1 signaling pathway in melanoma cells, confirming that Nobiletin is a promising therapeutic target for melanoma. In addition, a review article by Zhao et al. summarized the mechanism of ferroptosis in hepatocellular carcinoma and described recent advances in the treatment of drug resistance, demonstrating that multiple genes or compounds can sensitize sorafenib. Moreover, the preparation of nanoparticles such as MMSN and LDL-DHA in the tumor microenvironment and exosomes with ferroptosis induction can induce ferroptosis in HCC, thus effectively treating patients.

In conclusion, ferroptosis is associated with various pathophysiological processes and diseases, including cancer. Cancer cells require more iron than normal non-cancer cells, indicating that cancer cells are more susceptible to iron-dependent cell death. Therefore, inducing ferroptosis is a promising cancer treatment strategy that can effectively overcome resistance to conventional cancer treatments. This study provides evidence for the important role of ferroptosis in cancer treatment and suggests that many ferroptosis-related genes and molecules can be used as potential biomarkers for treatment and prognosis. Although ferroptosis has great advantages in cancer treatment, accurately determining clinical biomarkers related to ferroptosis, how to simply and accurately predict *in vivo* biomarkers of patients, and screening suitable ferroptosis inducer treatment are all problems that we need to solve in the future. In addition, cancer cells and immune cells have different internal mechanisms for ferroptosis, and we need to understand how they contribute to vulnerability to ferroptosis. More importantly, a subset of GPX4 inhibitors has shown adverse effects in animal models, which need to be addressed in ferroptosis targeted cancer therapies.
